# Supplementary material for: Perovskite multifunctional logic gates via bipolar photoresponse of single photodetector
Source: Nat Commun. 2022 Feb 7;13:720. doi: 10.1038/s41467-022-28374-w (PMC8821588; doi:10.1038/s41467-022-28374-w)
Supplement: Supplementary file 1 — Supplementary Information [file 41467_2022_28374_MOESM1_ESM.pdf]

Supplementary Information

Perovskite Multifunctional Logic Gates via Bipolar Photoresponse of Single Photodetector

Woochul Kim[1,2†], Hyeonghun Kim[2,5,†], Tae Jin Yoo[3], Jun Young Lee[1,6], Ji Young Jo[1], Byoung Hun Lee[3], Assa Aravindh Sasikala[4], Gun Young Jung[1,*], Yusin Pak[2,*]

[1]School of Materials Science and Engineering (SMSE), Gwangju Institute of Science and Technology (GIST), Gwangju 61005, Republic of Korea

[2]Sensor System Research Center, Korea Institute of Science and Technology (KIST), Seoul 02792, Republic of Korea

[3]Department of Electrical Engineering, Pohang University of Science and Technology, Gyeongbuk 37673, Republic of Korea

[4]Nano and molecular systems research unit (NANOMO), University of Oulu, Oulu 90750, Finland

[5]School of Engineering Technology, Purdue University, West Lafayette, IN 47907, USA

[6]Electronic Materials Research Center, Korea Institute of Science and Technology (KIST), Seoul 02792, Republic of Korea

*Correspondence to gyjung@gist.ac.kr, yusinpak@kist.re.kr

†These authors contributed equally to this work.

Supplementary Figures

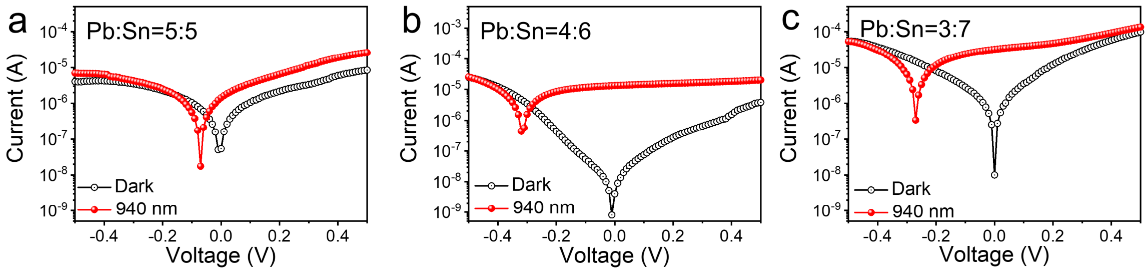

Supplementary Fig. 1 Optoelectronic properties of $MA_{0.5}FA_{0.5}Pb_xSn_{1-x}I_3$ depending on the cationic ratio. a-c *I-V* curves of $MA_{0.5}FA_{0.5}Pb_xSn_{1-x}I_3$ perovskite photodiodes with various ratios of Pb:Sn (**a** 5:5, **b** 4:6 ,and **c** 3:7) under dark and 940 nm illumination. In terms of photocurrent at zero bias, open-circuit voltage, and dark current, the best device performance was revealed at the Pb:Sn ratio of 4:6.

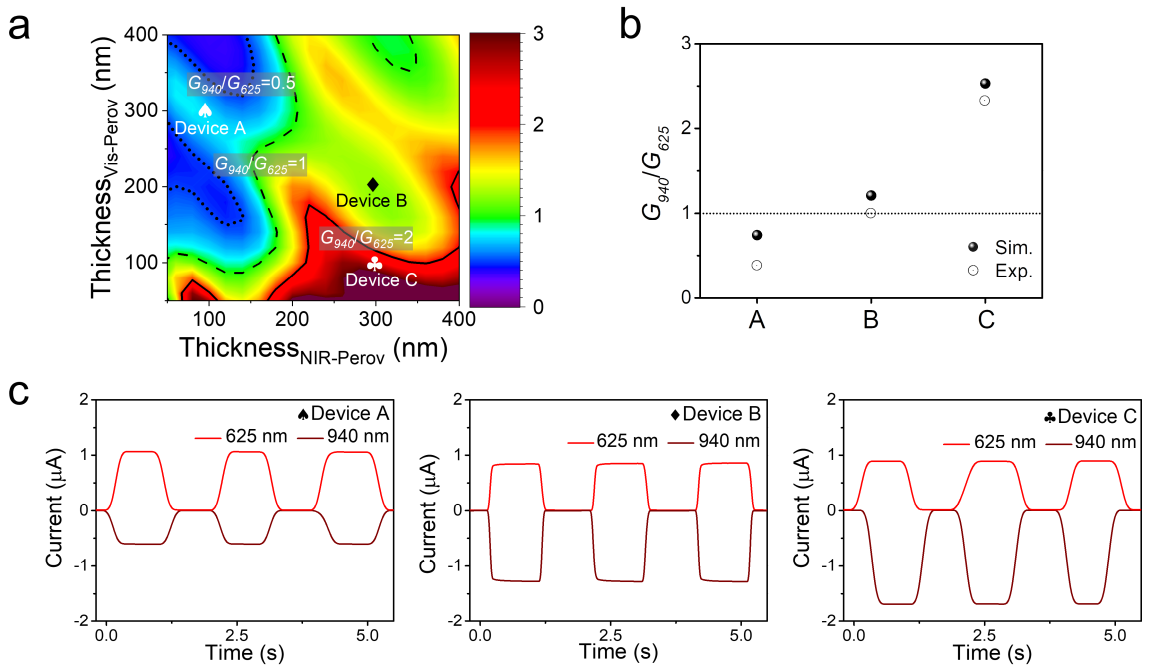

Supplementary Fig. 2 Investigation on optimized perovskite film thickness. a Simulation of photoconductive gain ratio (G_{940}/G_{625}) with different perovskite thicknesses. **b** Empirical and theoretical gain ratio values of the three devices; marked in **a**. **c** Steady-state currents of the device A, B, and C under 625 and 940 nm illumination.

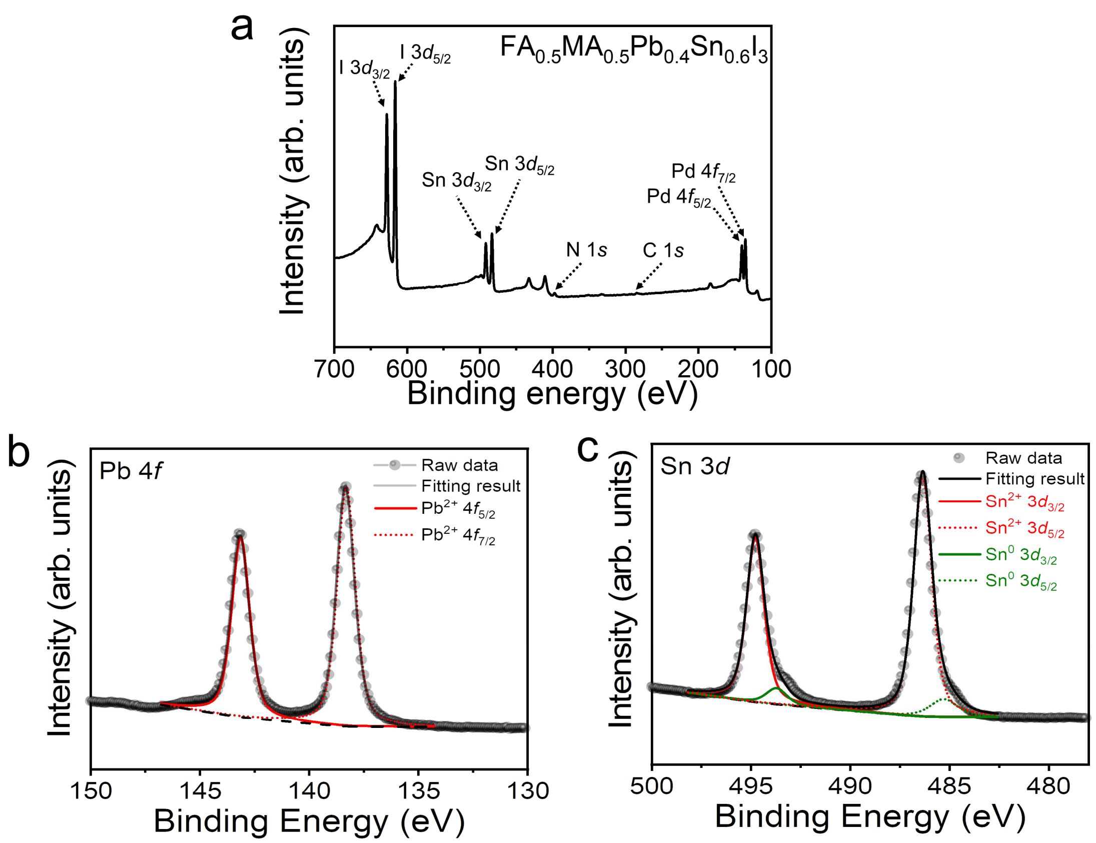

Supplementary Fig. 3 XPS analysis of $MA_{0.5}FA_{0.5}Pb_{0.4}Sn_{0.6}I_3$ perovskite film. a Wide XPS spectrum. **b**, **c** Narrow XPS spectra representing Pb 4*f* and Sn 3*d*.

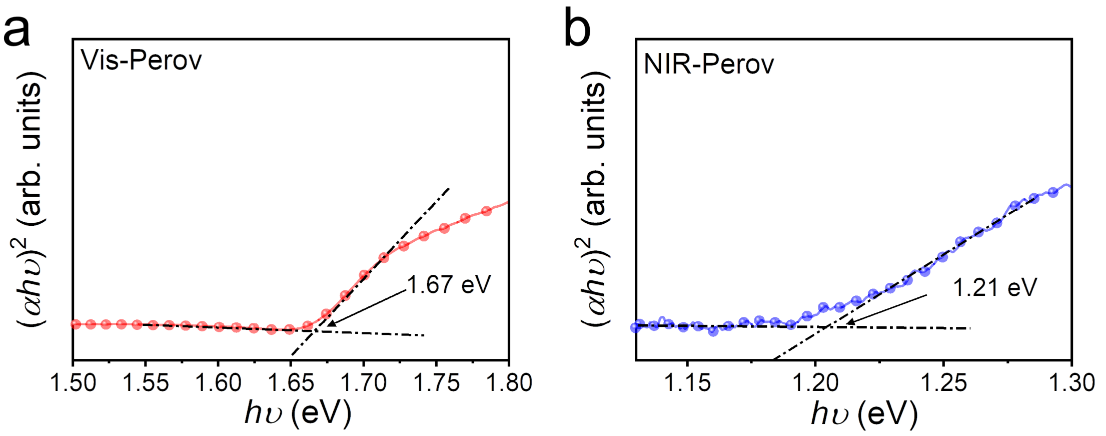

Supplementary Fig. 4 Optical bandgaps of perovskites. a, **b** Tauc plots converted from the absorption spectra of $MAPbI_3$ (Vis-Perov) and $MA_{0.5}FA_{0.5}Pb_{0.4}Sn_{0.6}I_3$ (NIR-Perov) perovskite films. The bandgaps of Vis-Perov and NIR-Perov were identified as 1.67 and 1.21 eV, respectively.

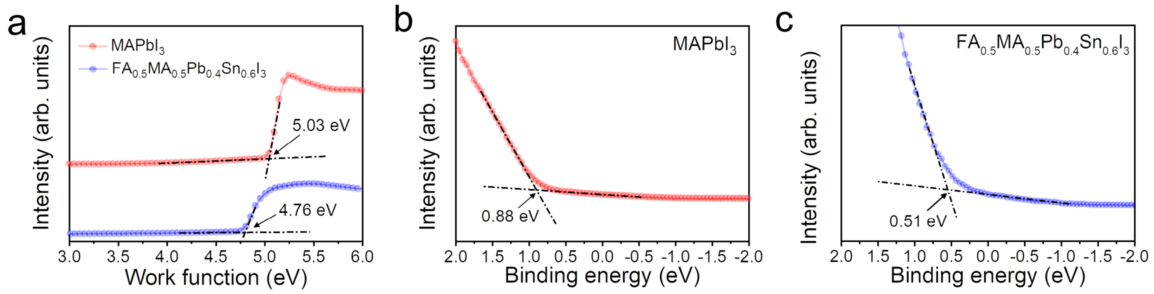

Supplementary Fig. 5 UPS analysis of perovskites. a Secondary electron cutoff of $MAPbI_3$ (red) and $MA_{0.5}FA_{0.5}Pb_{0.4}Sn_{0.6}I_3$ (blue) perovskite thin films prepared on Au-coated Si substrate. **b, c** Narrow-range binding energy spectra of $MAPbI_3$ and $MA_{0.5}FA_{0.5}Pb_{0.4}Sn_{0.6}I_3$.

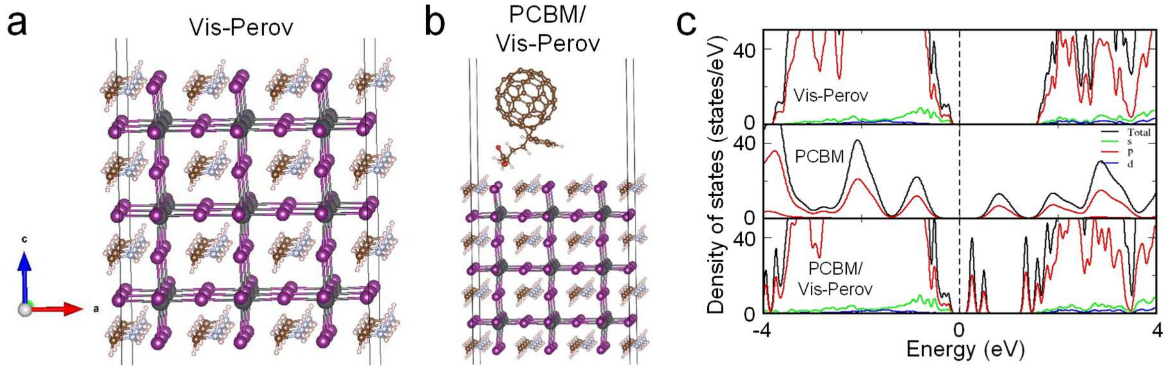

Supplementary Fig. 6 DFT calculation results of PCBM/Vis-Perov interface. a The optimized geometry of Vis-Perov surface. The colour codes of atoms are Pb–black, I–purple, C–brown, N–light blue, H–light pink, and O–red, respectively. **b** The optimized geometry of the perovskite surface when absorbing a PCBM molecule. **c** The density of states of Vis-Perov (top panel), PCBM molecule (middle panel), and PCBM/Vis-Perov interface (bottom panel).

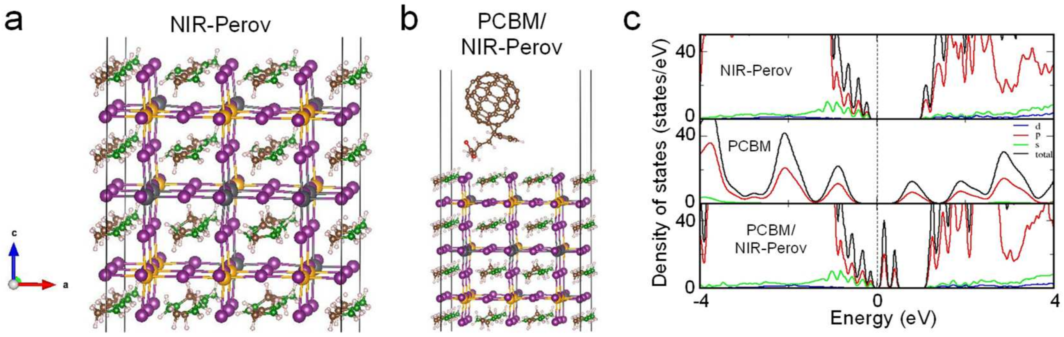

Supplementary Fig. 7 DFT calculation results of PCBM/NIR-Perov interface. a The optimized geometry of NIR-Perov. The colour codes of the atoms are Pb–black, I–purple, C–brown, N–light blue, H–light pink, O–red, and Sn–yellow, respectively. **b** The optimized geometry of the NIR-Perov surface when absorbing a PCBM molecule. **c** The density of states of NIR-Perov (top panel), PCBM molecule (middle panel), and PCBM/NIR-Perov interface (bottom panel).

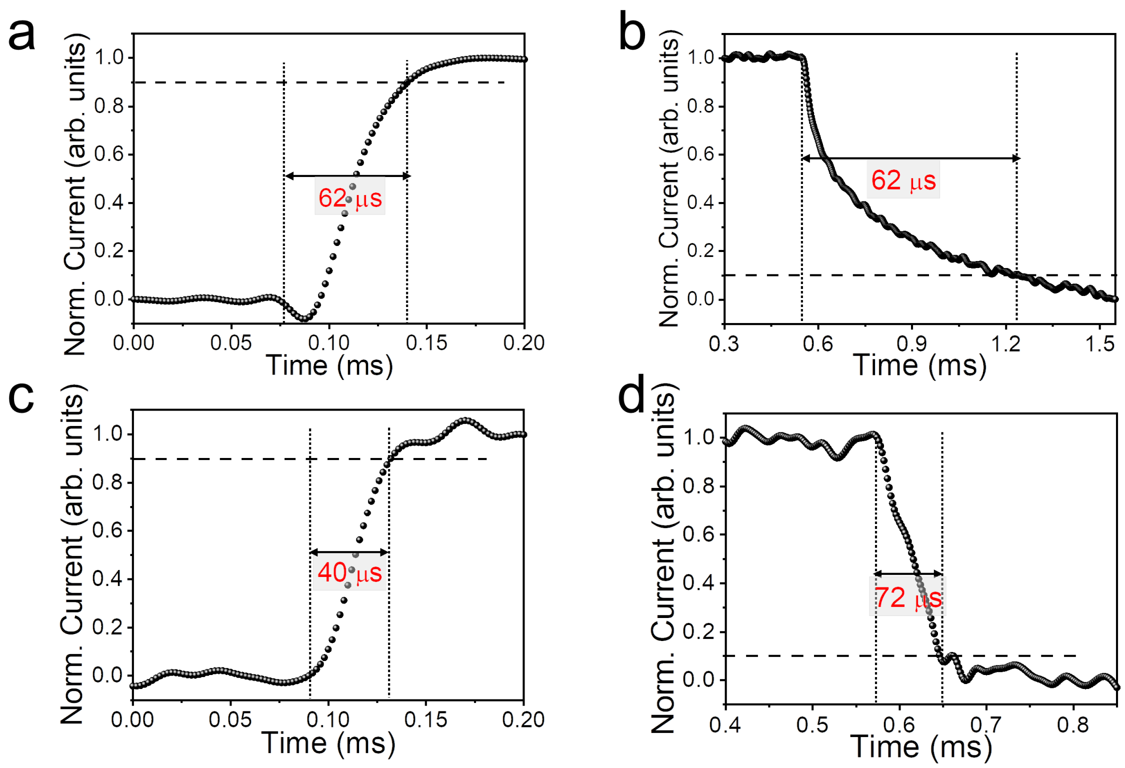

Supplementary Fig. 8 Operating speed of the SPPD with an active area of 0.04 mm^2. a, **b** Rise and decay times under 530 nm light illumination at the irradiance of 1 mW cm^{-2}. **c**, **d** Rise and decay times under 940 nm light illumination at the same irradiance. The rise time was defined as the time required for the current to reach 90 % of the saturation level under light illumination, while the decay time was the delayed time required to fall to 10 % of the saturation level.

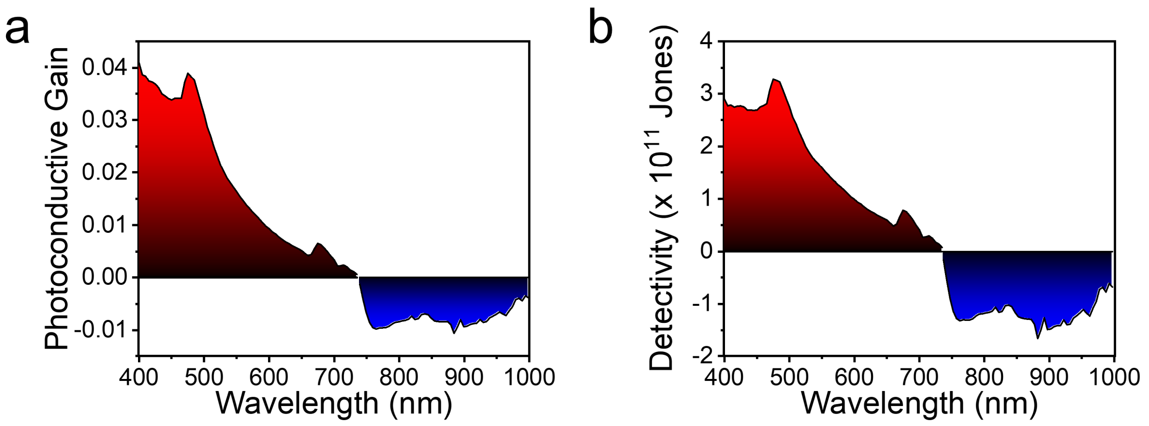

Supplementary Fig. 9 a Photoconductive gain and **b** specific detectivity of the SPPD under wavelength from 400 to 1000 nm.

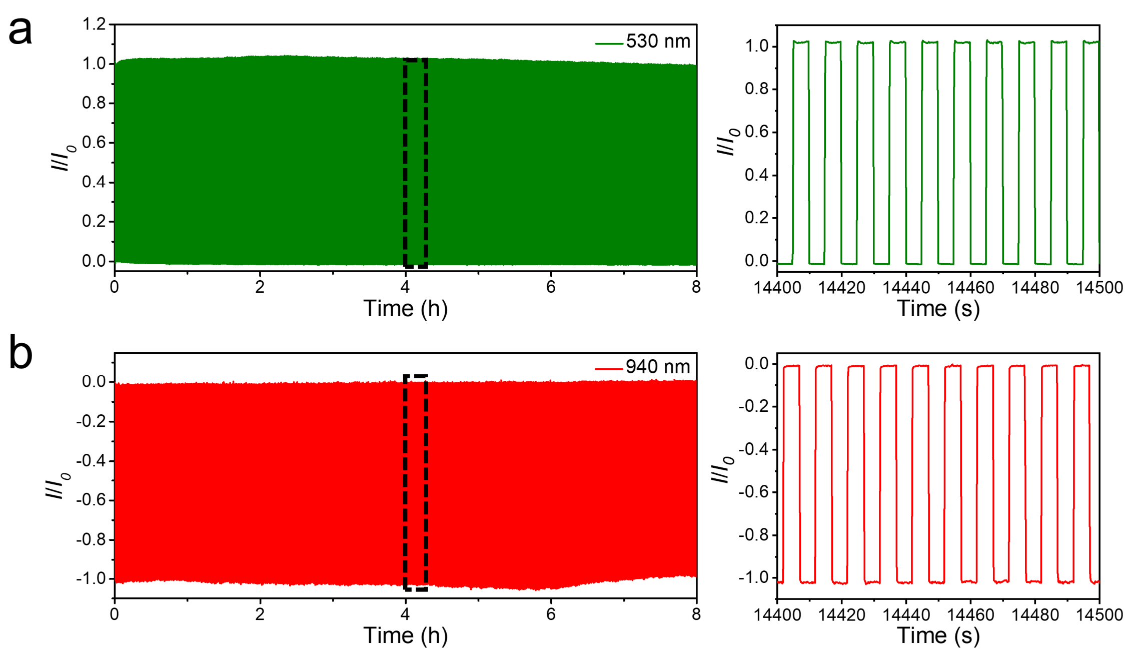

Supplementary Fig. 10 Repeatability tests of the SPPD. a, **b** Steady-state currents under light pulses (5 s on and 5 s off) at a wavelength of **a** 530 nm and **b** 940 nm. The measurements were conducted for 3000 pulses under ambient conditions, and the right inset figures represent the magnified current behavior after 4 hrs. The current values for each figure were normalized with respect to the photocurrent recorded at the first pulse.

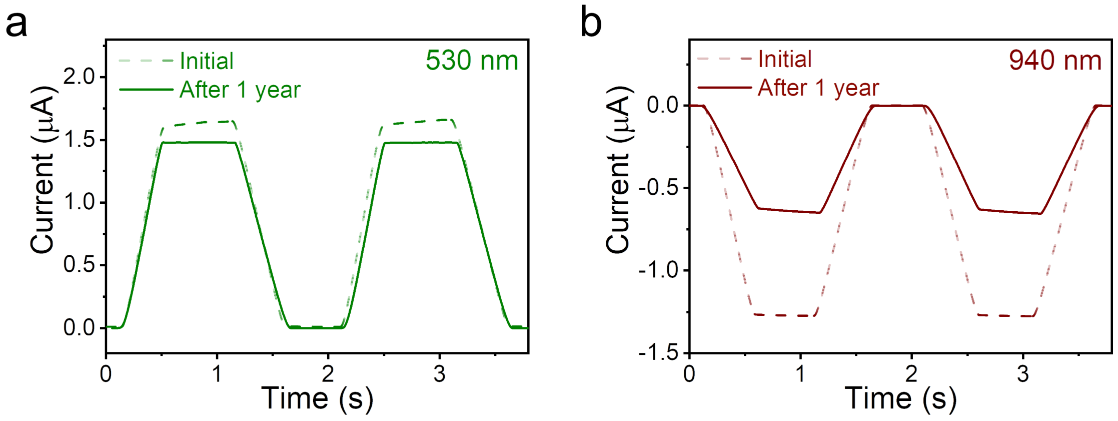

Supplementary Fig. 11 Long-term stability of the SPPD. a, **b** Steady-state currents under illumination of **a** 530 nm and **b** 940 nm lights after 1 year under dry-air condition.

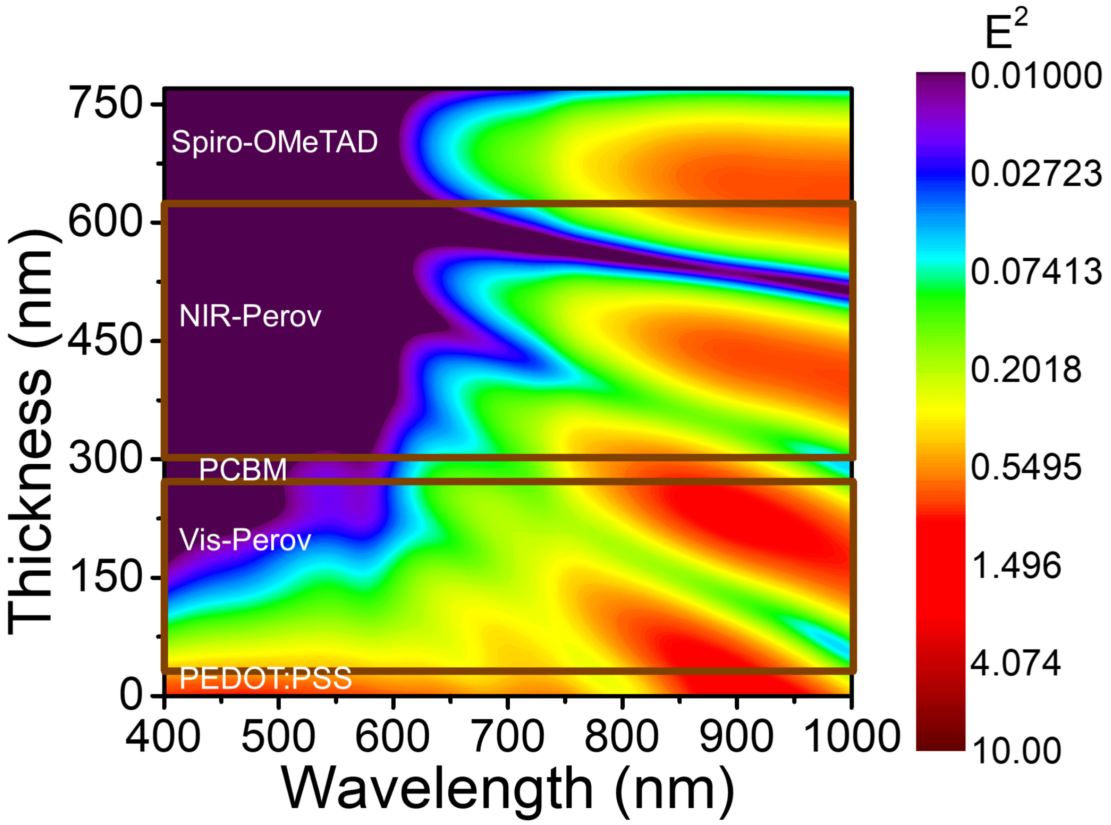

Supplementary Fig. 12 Simulation of the optical field (E^2) profile with respect to full device position of SPPD under monochromatic light illumination at wavelengths from 400 to 1000 nm.

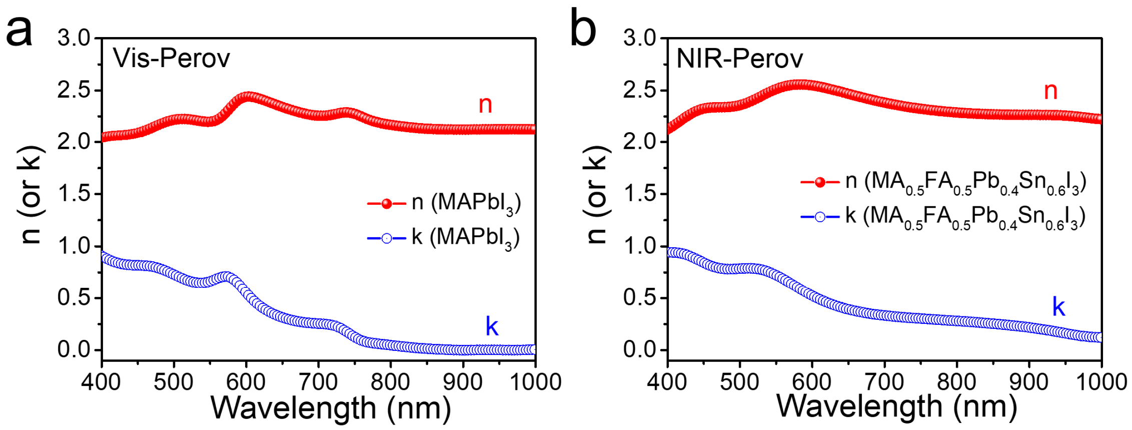

Supplementary Fig. 13 Optical parameters of perovskites for simulating optical field. a, **b** Complex refractive indices (n + ik) of the Vis-Perov and NIR-Perov that were obtained using an ellipsometer. Complex refractive indices for the other layers (ITO[10], Au[11], PEDOT:PSS[12], PCBM[13], and Spiro-OMeTAD[14]) were gathered through literature survey.

a

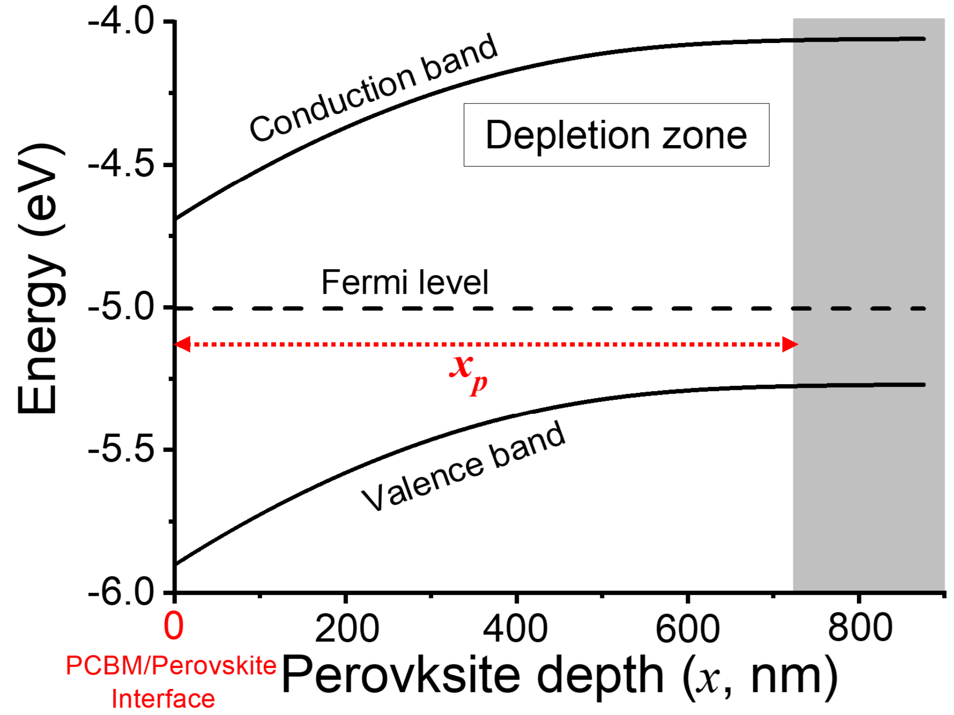

b

	V_i	N_a (or N_d) (cm^{-3})	ε	x_p
Vis-Perov	0.66	1.00×10^{12}	$10\varepsilon_0$	>10 μm
NIR-Perov	0.39	2.40×10^{15}	$24\varepsilon_0$	~220 nm
PCBM	-	1.92×10^{12}	$3.9\varepsilon_0$	

Supplementary Fig. 14 Energy band bending of perovskite in the SPPD. a Schematic of band bending of a perovskite. **b** Inherent parameters for each layers in the SPPD; V_i is the relative Fermi-level difference with the PCBM; N_a (N_d) is the hole (electron) concentration; ε is the dielectric constant (ε_0 is the vacuum permittivity); and x_p is the theoretical depletion width.

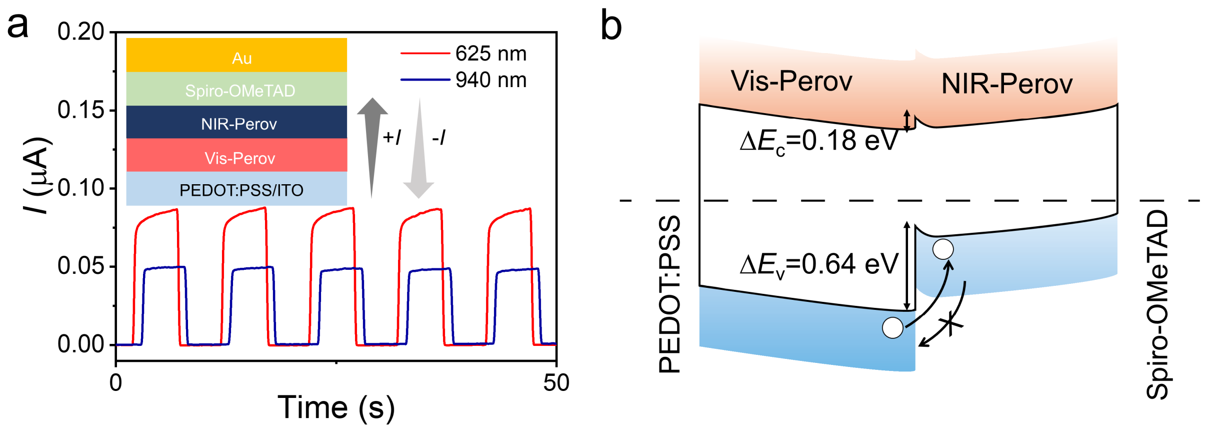

Supplementary Fig. 15 Optoelectronic properties of the PCBM-less PD. **a** Steady-state currents of the PCBM-less device under 625 and 940 nm irradiation (1 mW cm^{-2}). **b** Energy band diagram of the PCBM-less PD.

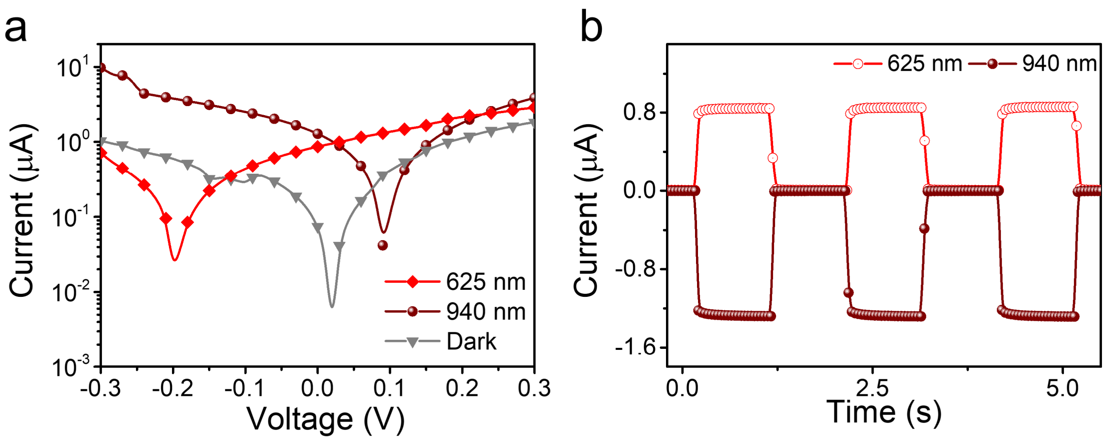

Supplementary Fig. 16 Optoelectronic properties of the SPPD. a, b *I*-*V* curves and steady-state currents at zero bias under dark and irradiation conditions (625 and 940 nm at 1 mW cm^{-2}).

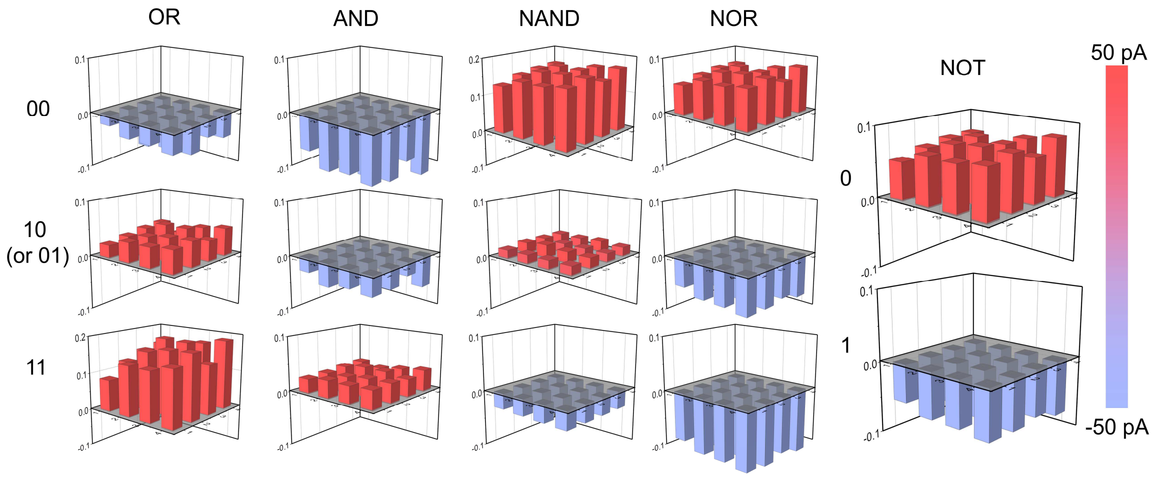

Supplementary Fig. 17 Logic gate operation of the 50×50 μm^2 pixel array using a pair of 530 and 940 nm light sources. Three-dimensional bar charts for all the outputs ("OR", "AND", "NAND", "NOR", and "NOT") obtained from the 16 pixels in SPPD-OELG array.

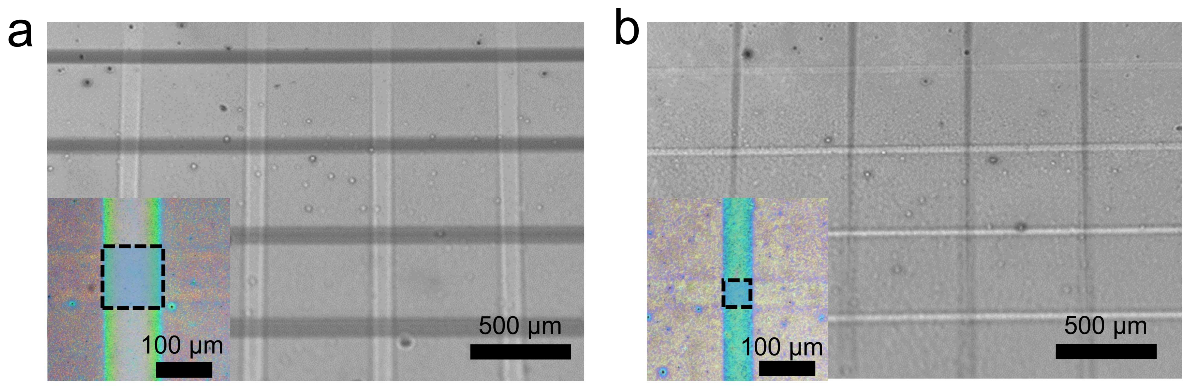

Supplementary Fig. 18 Optical microscope image of the 4×4 arrays with the pixel size of **a** 100×100 μm^2 and **b** 50×50 μm^2, respectively.

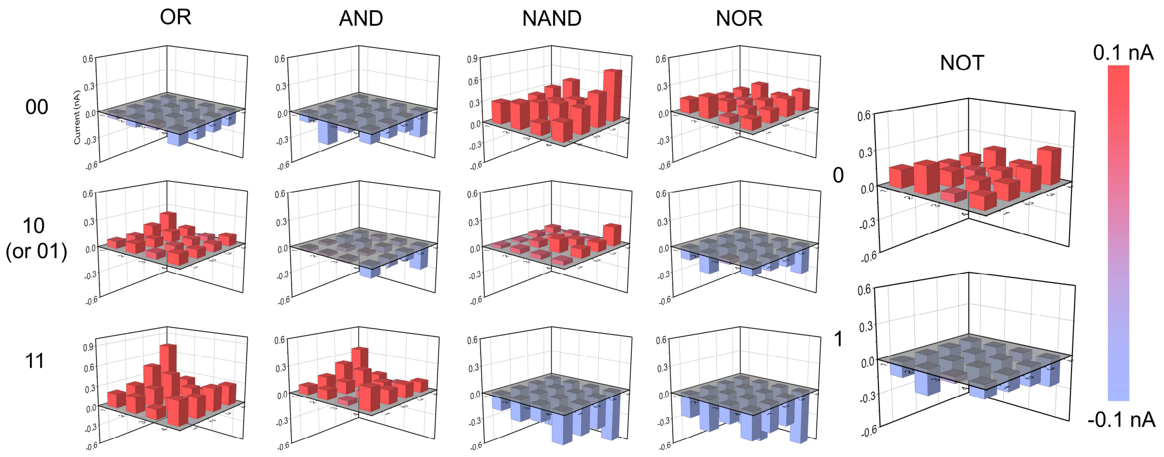

Supplementary Fig. 19 Logic gate operation of the 100×100 μm^2 pixel array. Three-dimensional bar charts for all the outputs ("OR", "AND", "NAND", "NOR", and "NOT") obtained from the 16 pixels.

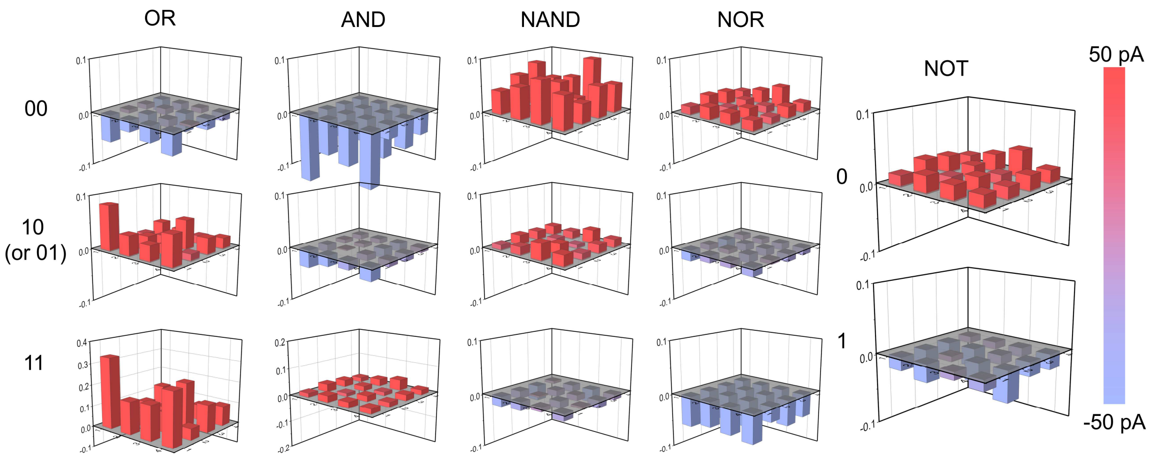

Supplementary Fig. 20 Logic gate operation of the 50×50 μm^2 pixel array. Three-dimensional bar charts for all the outputs ("OR", "AND", "NAND", "NOR", and "NOT") obtained from the 16 pixels.

Supplementary Tables

Supplementary Table 1 Comparison of the Hall values (carrier mobility and concentration) of Pb and Sn based perovskites.

Materials	Mobility ($cm^2V^{-1}s^{-1}$)	Carrier concentration (cm^{-3})	Reference
$MA_{0.5}FA_{0.5}Pb_{0.4}Sn_{0.6}I_3$ (NIR-Perov)	27	2.4×10^{15}	This work
$MAPbI_3$ (Vis-Perov)	-	$\sim10^{12}$	This work
$FASnI_3$	5–40	8×10^{15}–7×10^{16}	21
$FASnI_3$	19	2.4×10^{16}	22
$MAPbI_3$	15.9	1.02×10^{12}	23
$MAPbI_3$	58.79	3.375×10^{11}	24

Supplementary Table 2 Comparison of the rise and decay time of vertical perovskite-based photodetectors.

Materials	Structure	Bias (V)	Spectral region (nm)	Rise/Decay time (μs)	Ref.
Triazine-Th-OMeTAD/$MAPbI_3$/PCBM	Thin films	0	300 ~ 780	0.018/0.1	25
PTAA/$MAPbI_3$/FBIC/C_{60}	Thin films	-0.3 ~ 1.2	300 ~ 1000	35/20	26
m:F-2/MAPbI3/C_{60}	Thin films	0.78 ~ 0.90	400 ~ 780	0.0398	27
PEDOT:PSS/ Rb-$MAPb_xSn_{1-x}I_3$/PCBM	Thin films	0.01	300 ~ 1100	0.09/2.27	28
PEDOT:PSS/$MAPbI_3$/C_{60}	Thin films	0.1	300 ~ 800	0.12	29
PEDOT:PSS/ $MAPbI_3$/PCBM	Thin films	0	400 ~ 750	1.7/10.3	30
PEDOT:PSS/$CyBF_4$-$FA_{0.83}Cs_{0.17}Pb(I_{0.9}Br_{0.1})_3$/ PCBM	Thin films	0.5	400 ~ 1800	65/74	31
TiO_2/$Cs_{0.05}FA_{0.83}MA_{0.17}Pb(I_{0.9}Br_{0.1})_3$/Spiro-OMeTAD	Thin films	0	300 ~ 750	19/21	32
PTAA/$MAPbI_3$/ PDPPTDTPT/BCP	Thin films	0.2	400 ~ 1000	107/5	33
PCBM/$MAPbBr_{2.5}Cl_{0.5}$-$MAPbBr_3$-$MAPbI_3$/Au	Thin films	150	400 ~ 800	12.23	34
PEDOT:PSS/ $MAPbI_{3-x}Cl_x$/PCBM	Thin films	0 ~ 2	400 ~ 800	0.18/0.16	35
$MAPbCl_3$	Single crystals	0	300 ~ 400	0.015	36
PTAA/$MAPbI_3$/C_{60}	Thin films	0	400 ~ 800	0.0065	37
$MAPbI_3$/ $MA_{0.5}FA_{0.5}Pb_{0.4}Sn_{0.6}I_3$	Thin films	0	300 ~ 1000	60/680 @ 530 nm 40/72 @ 940 nm	This work

Supplementary Table 3 Comparison of the on-off ratio of optical logic gate (OLG) and optoelectronic logic gate (OELG).

Type	Materials	Logic type	On-off ratio (dB)	Ref.
OLG	Silicon (Si)	OR, XOR, NOT, XNOR, NAND	20	38
OLG	Graphene	AND, XNOR, NOR	18	39
OLG	Au metamaterials	OR, NOT,XNOR	24	40
OLG	Au metamaterials	AND, OR, NOT	9.4 ~ 28	41
OLG	InP-AlGaAs nanowires	NAND, NOR	<10	42
OELG	ZnO/GaN nanowires	AND	<6	43
OELG	BiVO4 film	OR	<15	44
OELG	Si-waveguide-integrated carbon nanotube (CNT)	OR, NOT	Inverted type Gain >41.7	45
OELG	CNT-Si junction	AND	40	46
OELG	Perovskite back-to-back diode	AND, OR, NAND, NOR, NOT	Theoretically infinite (polarity change)	This work

Supplementary Notes

Supplementary Note 1. | Optimized perovskite film thickness.

Before the device fabrication, we simulated theoretical photoconductive gain (G_λ) by varying the thickness of perovskite films to explore an optimized condition. As shown in Supplementary Fig. 2a, the color and tone represent the gain ratio (G_{940}/G_{625}), which is distributed on the plane as a function of perovskite film thickness ranging from 50 to 400 nm. The gain ratio can be obtained using the formula below:

$$\frac{\int_{T_{NIR-Perov}} \frac{hc}{925} C_R(940,x)dx}{\int_{T_{Vis-Perov}} \frac{hc}{625} C_R(625,x)dx - \int_{T_{NIR-Perov}} \frac{hc}{625} C_R(625,x)dx} \tag{1}$$

,where h and c are Planck constant and speed of light, respectively; $T_{NIR\text{-}Perov}$ and $T_{Vis\text{-}Perov}$ represent the thickness of NIR-Perov and Vis-Perov, respectively; $C_R(\lambda,x)$ is the charge generation rate at a certain depth position x of device under illumination of λ nm light source with unit irradiance (1 mW cm^{-2}).

Our logic gate works based on the offset of opposite photocurrents generated under the two light sources. Therefore, the logic gate operation becomes more efficient and controllable when the gain ratio becomes 1. The thickness condition (the combination of 300 nm NIR-Perov and 200 nm Vis-Perov) used for our logic gate devices ('Device B') locates within the optimized green zone (diamond symbol) at which the gain ratio is ~1.

A validation test was performed by comparing the photoresponses of three devices (denoted 'Device A', 'Device B' and 'Device C') and their photoconductive gain ratios

were investigated under 625 and 940 nm light illumination. Under the two light sources, the photoresponses of both 'Device A' and 'Device C', having a perovskite layer thickness less than 150 nm, are not as balanced as that of 'Device B', which is not good for the logic gate operation. The combination of 300 nm NIR-Perov and 200 nm Vis-Perov has the gain ratio of 1, optimal for the efficient logic gate operation.

Supplementary Note 2. | XPS analysis of $MA_{0.5}FA_{0.5}Pb_{0.4}Sn_{0.6}I_3$ perovskite film.

The wide XPS spectrum revealed a series of sharp peaks corresponding to core levels of I 3*d*, Sn 3*d*, and Pb 4*f*, while the peaks for N 1*s* and C 1*s* were weak or negligible. This was due to the degradation of the organic component during argon (Ar) etching process for XPS sample preparation. Judging from the peak area of Sn 3*d* and Pb 4*f* (Supplementary Fig. 3a), the atomic ratio of Pb:Sn was estimated as 4:6, which is coincided with that of the deposition rates for PbI_2 and SnI_2. The peak location of Pb 4*f* doublet (138 and 143 eV for $4f_{5/2}$ and $4f_{7/2}$, respectively, Supplementary Fig. 3b) was exactly consistent with that for the general Pb^{2+}. The Sn 3*d* doublet ($3d_{3/2}$ and $3d_{5/2}$, Supplementary Fig. 3c) were de-convoluted into two peaks corresponding to Sn^{2+} and metallic Sn^0 states. The over-oxidized Sn ($Sn^{2+}\rightarrow Sn^{4+}$), which impedes the generation of photo-induced charges, was not observed, indicating the suppression of undesirable oxidation within the perovskite film.

Supplementary Note 3. | UPS analysis of perovskites.

The UPS spectra determined the work functions: 5.03 and 4.76 eV for $MAPbI_3$ and $MA_{0.5}FA_{0.5}Pb_{0.4}Sn_{0.6}I_3$, respectively. The onset of the narrow-range binding energy spectra corresponded to the difference between Fermi level and valence band maximum, which were measured as 0.88 eV for $MAPbI_3$ and 0.51 eV for $MA_{0.5}FA_{0.5}Pb_{0.4}Sn_{0.6}I_3$.

Supplementary Note 4. | Density functional theory (DFT) calculation.

To corroborate the experimental results, density functional theory (DFT) calculations were carried out using the plane wave pseudopotential code, Vienna Abinitio Simulation Package (VASP)[1,2]. We exploited the generalized gradient approximation (GGA) within the Perdew Burke Eizenhoff (PBE) scheme[3] to account for the exchange and correlation interactions. The kinetic energy cutoff for the plane waves, included in the basis set, was chosen as 650 eV after convergence tests, while the convergence criterion for electronic self-consistent loop was 10^{-6} eV with a force tolerance of 0.001 eV $Å^{-1}$. The structures were relaxed with the conjugate-gradient algorithm. The valence electrons, included in calculations for the atoms, were as follows; oxygen (O)=6, carbon (C)=4, hydrogen (H)=1, nitrogen (N)=5, lead (Pb)=4, iodine=7, and tin (Sn)=4[4].

1) DFT calculation results of PCBM/Vis-Perov interface.

The lattice parameters were set as a=6.463 Å, b=6.415 Å, and c=6.516 Å, which were coincided to those of the most stable perovskite surface reported in literature[5,6]. Furthermore, we considered the methyl ammonium terminated surfaces as they possess more stability in the (001) orientation, compared to other orientations[6]. The optimized structure of Vis-Perov before and after PCBM adsorption are shown in Supplementary Fig. 6a and b, respectively. The surfaces were simulated using a 3x3x3 supercell, while vacuum layer of 25 Å thickness was added along the c axis to minimize the interaction between the periodic slabs. A Monkhorst Pack K grid of 3x3x1 was employed for the Brillouin zone integration. The density of states (DOS) for the Vis-Perov, PCBM and PCBM/Vis-Perov interface are shown in top, middle and lower

panels in Fig. 6c. One significant finding is that the PCBM adsorption causes an emergence of additional energy states between the conduction and valence band of Vis-Perov.

2). DFT calculation results of PCBM/NIR-Perov interface

The NIR-Perov was modeled by replacing formamidinium (FA) ions with methylammonium (MA) ions as well as Pb with Sn to match the experimental concentrations. The optimized structures were stable with respect to the mixing of ions and molecules and the optimized geometries are shown in Supplementary Fig. 7a and b, respectively. We considered the Van der Waals interaction in the calculations as implemented in VASP[7] to account for the dispersion effects. The orbital-resolved DOS distribution of the NIR-Perov, PCBM and PCBM/NIR-Perov interface is presented in Fig. 7c. The obtained bandgap for the NIR-Perov (1 eV) was relatively narrow, compared to that of Vis-Perov (1.6 eV). Furthermore, in the same manner as the Vis-Perov, new energy states emerged in the band structure of the NIR-Perov as the PCBM is attached to the top surface. Considering the energy level of the DOS of PCBM, which appeared below the conduction band for the NIR-Perov, electron transfer from the PCBM to NIR-Perov could be facilitated, thus resulting in a high-performance photo-response even at zero bias.

Supplementary Note 5. | Investigation of photoconductive gain and detectivity.

We calculated photoconductive gain (G_λ) and specific detectivity (D_λ) using the following formulas adopted in the literatures[8,9]

$$G_\lambda = \frac{R_\lambda \cdot h \cdot c}{q \cdot \lambda} \tag{2}$$

$$D_\lambda = \frac{R_\lambda}{\sqrt{2 \cdot q \cdot I_\mathrm{d} \cdot A^{-1}}} \tag{3}$$

,where R_λ is a responsivity of photodetector, recorded under a wavelength of light of λ. , I_d and A are a dark current and active area of the device, respectively; h, c, and q are physical constants representing Planck constant, speed of light, and unit charge, respectively. Note that we employed specific detectivity (D_λ), with the assumption that a shot noise dominantly determines a noise current of photodetector. The calculated photoconductive gains under 530 nm, 625 nm, and 940 nm light illumination were 0.02, 0.007 and 0.008, respectively. The corresponding detectivities were calculated as 1.89, 0.76, and 1.27×10^{11} Jones, respectively.

Supplementary Note 6. | Repeatability test.

We conducted the repeatability for the SPPD under 530 and 940 nm light pulses (5s on / 5s off). The photocurrent values are normalized with respect to the photocurrent recorded at the first pulse. Each magnified photocurrent behavior recorded after 4 hrs clearly shows the on-off current behavior without degradation. In this test, the SPPD retained its initial responsivity even after 8 hrs (approximately 3000 pulse pulses) under ambient conditions.

Supplementary Note 7. | Long-term stability.

For long-term stability testing, we analyzed another SPPD that had been stored in laboratory for one year under dry-air condition. The saturated on-current, recorded under 530 and 940 nm lights, was reduced to 90% and 50% of the initial value, respectively.

Supplementary Note 8. | Band alignments of the SPPD.

A built-in potential caused by a Fermi-level difference leads to band-bending at material interfaces[15-17], as shown in Supplementary Fig. 14a. Herein, we may ignore the band-bending at the interfaces between the perovskites and hole transport layers (PEDOT:PSS or Spiro-OMeTAD) because of the minute Fermi-level difference. Then, we can simply consider the heterojunction between the PCBM and perovskite regarding on this matter. The built-in potential across the perovskite can be estimated as

$$V(x) = -V_i + \frac{qN_a}{2\varepsilon_{\mathrm{Perov}}}(x - x_p)^2 \tag{4}$$

,where V_i is the Fermi-level difference between the PCBM and perovskite; $\varepsilon_{\mathrm{Perov}}$ and N_a are the dielectric constant and doping concentration of perovskite, respectively; q is the unit charge; x is the perovskite depth from the interface[15-17]; and x_p is the thickness of depletion zone (energy-bending region) and can be defined as

$$x_p = \left[\frac{2V_i \varepsilon_{\mathrm{PCBM}} \varepsilon_{\mathrm{Perov}} N_{\mathrm{d}}}{qN_a(\varepsilon_{\mathrm{PCBM}} N_{\mathrm{d}} + \varepsilon_{\mathrm{Perov}} N_a)}\right]^{1/2} \tag{5}$$

,where $\varepsilon_{\mathrm{PCBM}}$ and N_d are the dielectric constant and doping concentration of PCBM layer, respectively. We measured the hole concentration of NIR-Perov (2.40×10^{15} cm^{-3}) and Vis-Perov (1.00×10^{12} cm^{-3}) through Hall measurement. The other parameters (V_i and ε) were investigated through UPS (Supplementary Fig. 5) and literature survey[18-20]. All information on the perovskites and PCBM are summarized in Supplementary Fig. 14b. From the formula above, we confirmed that the theoretical depletion width (x_p) of Vis-Perov is above 10 μm which is much thicker than the actual

thickness (~200 nm). It implies that the film was fully depleted, rendering the inclined energy band through the entire film. This estimation can be well accepted considering the intrinsic Vis-Perov. The x_p of NIR-Perov was calculated as ~220 nm, which is more than two-thirds of the NIR-Perov thickness. Thus, the energy band of NIR-Perov is also expected to be bent across the film.

Supplementary Note 9. | Optoelectronic properties of the PCBM-less PD.

The PCBM layer serves as an intermediate *n*-type semiconductor playing a key role for realizing the bipolar photoresponse of p^+-*i*-*n*-*p*-p^+ device. To confirm the role of PCBM layer, we fabricated a PCBM-less device (the inset of Supplementary Fig. 15a). Both NIR and visible lights drove photocurrents in the same direction (Vis-Perov→NIR-Perov). The photocurrent was markedly suppressed to < 0.1 μA, 20 times smaller than that of the PCBM-interposed device.

The unidirectional photocurrent generation in the PCBM-less device can be explicated based on the energy band diagram depicted in Supplementary Fig. R15b. The large valence band offset (ΔE_V) between the two perovskites can influence hole transport. The deep HOMO level of the Vis-Perov, located at 0.64 eV lower than that of the NIR-Perov, facilitates hole transport from Vis-Perov to NIR-Perov, simultaneously hindering the reverse flow. Therefore, photo-generated holes are allowed to flow in only one direction regardless of incident wavelength.

Supplementary Note 10. | Logic gate operation of the 50×50 μm^2 pixel array.

The photoconductive gain for 530 nm light is 0.02, much larger than that (0.007) for the 625 nm. In this case, the irradiance of 530 nm, equivalent to a third of that of the 625 nm, should be used to achieve optimal logic outputs. Then, the five logics were faithfully implemented in the same SPPD device under 530 and 940 nm illumination.

Supplementary Note 10. | Hall measurements of the perovskite films.

From the Hall measurements, we confirmed that the NIR-Perov has a mobility of 27 $cm^2V^{-1}s^{-1}$ and a hole concentration of 2.4×10^{15} cm^{-3}, which are very similar to the reported values (*p*-type Sn-based perovskites)[21,22]. Considering the location of Fermi-level (Fig. 1d) and the hole concentration level of ~10^{15} cm^{-3}, the NIR-Perov is a lightly doped *p*-type semiconductor.

The carrier concentration of Vis-Perov was measured as ~10^{12} cm^{-3}, but the mobility was not reliably measured due to its low carrier concentration. We found in literature that intrinsic $MAPbI_3$ thin films had a concentration of 10^{11}–10^{12} cm^{-3} [23,24]. Therefore, the Vis-Perov film is regarded as an intrinsic semiconductor.

Supplementary references

1. Kresse, G. & Hafner, J. Ab *initio* molecular dynamics for liquid metals. *Phys. Rev. B* **47**, 558(R) (1993).

2. Kresse, G. & Joubert, D. From ultrasoft pseudopotentials to the projector augmented-wave method. *Phys. Rev. B* **59**, 1785 (1999).

3. Perdew, J. P., Burke, K. & Ernzerhof, M. Generalized grdient approximation made simple. *Phys. Rev. Lett.* **77**, 3865 (1996).

4. Kresse, G. & Hafner, J. Norm-conserving and ultrasoft pseudopotentials for first-row and transition elements. *J. Phys.: Condens. Matt.* **6**, 8245 (1994).

5. Quarti, C., Angelis, F. D. & Beljonne, D. Influence of surface termination on the energy level alignment at the $CH_3NH_3PbI_3$ perovskite/C_{60} interface. *Chem. Mater.* **29**, 958–968 (2017).

6. Quarti, C., Mosconi, E. & Angelis, F. D. Interplay of orentation order and electronic structure in methylammonium lead iodide: implications for solar cell operation. *Chem. Mater* **26**, 6557–6559 (2014).

7. Tkatchenko, A. & Scheffler, M. Accurate molecular Van Der Waals interactions from ground-state electron density and free-atom reference data. *Phys. Rev. Lett.* **102**, 073005 (2009).

8. Ma, N. et al. Photovoltaic–pyroelectric coupled effect induced electricity for self-powered photodetector system. *Adv. Mater.* **29**, 1703694 (2017).

9. Ma, N & Yang, Y. Enhanced self-powered UV photoresponse of ferroelectric $BaTiO_3$ materials by pyroelectric effect. *Nano Energy* **40**, 352-359 (2017).

10. Moerland, R. J. & Hoogenboom, J. P. Subnanometer-accuracy optical distance ruler based on fluorescence quenching by transparent conductors. *Optica* **3**, 112 (2016).

11. Chen, C. W. *et al.* Optical properties of organometal halide perovskite thin films and general device structure design rules for perovskite single and tandem solar cells. *J. Mater. Chem. A* **3**, 9152–9159 (2015).

12. Peumans, P., Yakimov, A. & Forrest, S. R. Small molecular weight organic thin-film photodetectors and solar cells. *J. Appl. Phys.* **93**, 3693–3723 (2003).

13. Bailie, C. D. *et al.* Semi-transparent perovskite solar cells for tandems with silicon and CIGS. *Energy Environ. Sci.* **8**, 956–963 (2015).

14. P. B. Johnson and R. W. Christy. Optical Constant of the Nobel Metals. *Phys. Rev. B* **6**, 4370–4379 (1972).

15. Guerrero, A. et al. Electrical field profile and doping in planar lead halide perovskite solar cells. *Appl. Phys. Lett.* **105**, 133902 (2014).

16. Crovetto, A. et al. How the relative permittivity of solar cell materials influences

solar cell performance. *Solar Energy* **149**, 145-150 (2017).

17. Moeini, I. et al. Modeling the time-dependent characteristics of perovskite solar cells. *Solar Energy* **170**, 969-973 (2018).

18. Maibach, J. et al. The band energy diagram of PCBM–DH6T bulk heterojunction solar cells: synchrotron-induced photoelectron spectroscopy on solution processed DH6T:PCBM blends and in situ prepared PCBM/DH6T interfaces. *J. Mater. Chem. C* **1**, 7635-7642 (2013).

19. Yang, Y. et al. Effect of doping of NaI monovalent cation halide on the structural, morphological, optical and optoelectronic properties of $MAPbI_3$ perovskite. *J. Mater. Sci: Mater. Electron.* **29**, 205-210 (2018).

20. Li, Y. et al. Highly conductive P-type $MAPbI_3$ films and crystals via sodium doping. *Font. Chem.* **8**, 754 (2020).

21. Tang, G. et al. Synergistic effects of the zinc acetate additive on the zinc acetate additive on the performance enhancement of Sn-based perovskite solar cells. *Mater. Chem. Front.* **5**, 1995-2000 (2021).

22. Liu, C.-K. et al. Sn-based perovskite for highly sensitive photodetectors. *Adv. Sci.* **6**, 1900751 (2019).

23. Yang, Y. et al. Effect of doping of NaI monovalent cation halide on the structural, morphological, optical and optoelectronic properties of $MAPbI_3$ perovskite. *J. Mater. Sci: Mater. Electron.* **29**, 205-210 (2018).

24. Shi, D. et al. Low trap-state density and long carrier diffusion in organolead trihalide perovskite single crystals. *Science* **347**, 519-522 (2015).

25. Shan, C. et al. An ultrafast-response and high-detectivity self-powered perovskite photodetector based on a triazine-derived star-shaped small molecules as a dopant-free hole transporting layer. *J. Mater. Chem. C* **9**, 7632 (2021).

26. Li, C. et al. Ultrafast and broadband photodetectors based on a perovskite/organic bulk heterojunction for large dynamic-range imaging. *Light Sci. Appl.* **9**, 31 (2020).

27. Zhang, D et al. Incorporating deep electron traps into perovskite devices: towards high efficiency solar cells and fast photodetectors. *J. Mater. Chem. A* **6**, 21039-21046 (2018).

28. Zhu, H. L. et al. Achieving high-quality Sn-Pn perovskite films on complementary metal-oxide-semiconductor-compatable metal/silicon substrates for efficient imaging array. *ACS Nano* **13**, 11800–11808 (2019).

29. Fang, Y. & Huang, J. Resolving weak light of sub-picowatt per square centimeter by hybrid perovskite photodetectors enabled by noise reduction. *Adv. Mater* **27**, 2804–2810 (2015).

30. Ma, Z. et al. High-performance self-powered perovskite photodetector for visible

light communication. *Appl. Phys. A* **126**, 869 (2020).

31. Lin, Q. et al. Near-infrared and short-wavelength infrared photodiodes based on dye-perovskite composites. *Adv. Func. Mater.* **27**, 1702485 (2017).

32. Adams, G. R et al. Fabrication of rapid response self-powered photodetector using solution-proceesed triple cation lead-halide perovskite. *Eng. Res. Express* **2**, 015043 (2020).

33. Shen, L. et al. Integration of perovskite and polymer photoactive layers to produce ultrafast response, ultraviolet-to-near-infrared, sensitive photodetectors. *Mater. Horiz.* **4**, 242 (2017).

34. Xu, Y. Perovskite photodetectors based on p-i-n junction with epitaxial electron-blocking layers. *Front. Chem.* **15**, 811 (2020).

35. Dou. L et al. Solution-processed hybrid perovskite photodetectors with high detectivity. *Nat. Commun.* **5**, 5404 (2014).

36. Chen, Z. et al. Solution-processed visible-blind ultraviolet photodetectors with nanosecond response time and high detectivity. *Adv. Opt. Mater.* **7**, 1900506 (2019).

37. Shen, L. et al. A self-powered, sub-nanosecond-response solution processed hybrid perovskite photodetector for time resolved photoluminescence-lifetime detection. *Adv. Mater.* **28**, 10794-10800 (2016).

38. Fu, Y., Hu, X. & Gong, Q. Silicon photonic crystal all-optical logic gates. *Phys. Lett. A* **377**, 329–333 (2013).

39. Rezaei, M. H., Zarifkar, A. & Miri, M. Ultra-compact electro-optical graphene-based plasmonic multi-logic gate with high extinction ratio. *Opt. Mater.* **84**, 572–578 (2018).

40. Fu, Y. *et al*. All-optical logic gates based on nanoscale plasmonic slot waveguides. *Nano Lett.* **12**, 5784−5790 (2012).

41. Peng, C. *et al*. Universal linear-optical logic gate with maximal intensity contrast ratios. *ACS Photonics* **5**, 1137−1143 (2018).

42. Yang, H. *et al*. Nanowire network-based multifunctional all-optical logic gates. *Sci. Adv.* **4**, eaar7954 (2018).

43. Bie, Y.-Q. *et al*. Self-powered, ultrafast, visible-blind UV detection and optical logical operation based on ZnO/GaN nanoscale p-n junctions. *Adv. Mater.* **23**, 649–653 (2011).

44. Zhao, J. *et al*. A Boolean OR gate implemented with an optoelectronic switching memristor. *Appl. Phys. Lett.* **115**, 153504 (2019).

45. Ma, Z. *et al*. Silicon-waveguide-integrated carbon nanotube optoelectronic system on a single chip. *ACS Nano* **14**, 7191−7199 (2020).

46. Kim, Y. L. *et al*. Voltage-switchable photocurrents in single-walled carbon nanotube-silicon junctions for analog and digital optoelectronics. *Nat. Photonics* **8**, 239−243 (2014).
